# Nitration of β-Lactoglobulin but Not of Ovomucoid Enhances Anaphylactic Responses in Food Allergic Mice

**DOI:** 10.1371/journal.pone.0126279

**Published:** 2015-05-08

**Authors:** Susanne C. Diesner, Cornelia Schultz, Chloé Ackaert, Gertie J. Oostingh, Anna Ondracek, Caroline Stremnitzer, Josef Singer, Denise Heiden, Franziska Roth-Walter, Judit Fazekas, Vera E. Assmann, Erika Jensen-Jarolim, Hanno Stutz, Albert Duschl, Eva Untersmayr

**Affiliations:** 1 Department of Pathophysiology and Allergy Research, Center of Pathophysiology, Infectiology and Immunology, Medical University of Vienna, Vienna, Austria; 2 Department of Pediatrics and Adolescent Medicine, Medical University of Vienna, Vienna, Austria; 3 Department of Molecular Biology, University of Salzburg, Salzburg, Austria; 4 Biomedical Sciences, Salzburg University of Applied Sciences, Puch/Salzburg, Austria; 5 Comparative Medicine, Messerli Research Institute of the University of Veterinary Medicine Vienna, Medical University Vienna and University Vienna, Vienna, Austria; Harvard Medical School, UNITED STATES

## Abstract

**Background:**

We revealed in previous studies that nitration of food proteins reduces the risk of *de novo* sensitization in a murine food allergy model. In contrast, in situations with preformed specific IgE antibodies, *in vitro* experiments suggested an increased capacity of effector cell activation by nitrated food proteins.

**Objective:**

The aim of this study was to investigate the influence of protein nitration on the effector phase of food allergy.

**Design:**

BALB/c mice were immunized intraperitoneally (i.p.) with the milk allergen β-lactoglobulin (BLG) or the egg allergen ovomucoid (OVM), followed by intragastric (i.g.) gavages to induce a strong local inflammatory response and allergen-specific antibodies. Subsequently, naïve and allergic mice were intravenously (i.v.) challenged with untreated, sham-nitrated or nitrated BLG or OVM. Anaphylaxis was monitored by measuring core body temperature and determination of mouse mast cell protease-1 (mMCP-1) levels in blood.

**Results:**

A significant drop of body temperature accompanied with significantly elevated concentrations of the anaphylaxis marker mMCP-1 were only observed in BLG allergic animals challenged with nitrated BLG and not in OVM allergic mice challenged with nitrated OVM. SDS-PAGE and circular dichroism analysis of the differentially modified allergens revealed an effect of nitration on the secondary protein structure exclusively for BLG together with enhanced protein aggregation.

**Conclusion:**

Our data suggest that nitration affects differently the food allergens BLG and OVM. In the case of BLG, structural changes favored dimerization possibly explaining the increased anaphylactic reactivity in BLG allergic animals.

## Introduction

Food allergies are on the rise and have become a significant health problem in Europe [1]. Proteins of cow’s milk and hen’s egg are among the most important food allergens in Europe triggering food allergies especially in children rather than adults. The overall self-reported lifetime prevalence is 6% for milk allergy and 2.5% for egg allergy being accompanied by high sensitization rates [2]. Patients often believe that elevated allergen specific IgE is synonymous with the existence of allergy. However, sensitization, which is defined as the existence of allergen specific IgE, can be clinically irrelevant as patients well tolerate the suspected food proteins. A variety of factors are discussed to influence sensitization and food allergy outcomes including sex, genetics, increased hygiene and diet [3]. In addition, chemical modifications of food proteins have been associated with altered immune responses [4–6]. Nitration of proteins, which is the addition of a nitro group to the aromatic ring of a tyrosine residue, has been discribed to increase allergenicity [7]. Pollutants, such as NOx and ozone [8], were discovered to chemically alter airborne proteins by nitration [7] affecting protein conformation and T-cell as well as B-cell epitopes [6,9]. In case of the major birch pollen allergen, Bet v 1a, nitration occurs with different propensity on individual tyrosine residues providing a mixture of nitration variants [10]. Moreover, Bet v 1a nitration influenced antigen presentation and processing via HLA-DR [11]. Nitrated Bet v1 is also associated with enhanced formation of oligomers, thereby delaying endolysosomal degradation. Additionally, a decreased production of Th1 and proinflammatory cytokines was observed in dendritic cells (DCs) potentially favouring a Th2 response [12]. Within the human body, endogenous nitration represents an important type of posttranslational protein modification occurring during inflammatory processes [13] or as a result of the aging process [14,15]. The extent of protein nitration in inflammatory responses was even suggested as a marker for oxidative stress in the human body [16]. Furthermore, diet derived nitrating agents such as nitrate and nitrite have been suggested to promote nitration in the gastrointestinal tract [17]. Protonation of nitrite in the acidic environment of the stomach is the first step for the formation of a variety of nitrating species, which might interact with proteins or allergens in the stomach [18]. In addition, food per se might already contain nitrated proteins [19], although the presence of nitrated tyrosine residues has not been investigated in depth [17]. Taken together, exogenous and endogenous nitrotyrosine (3-NT) formation could have an impact on food allergy. We recently investigated the influence of protein nitration on food allergy development in a food allergy mouse model. Nitrated ovalbumin (OVA), a major hen’s egg white allergen, increased sensitization only, when injected intraperitoneally (i.p.). In case of oral administration, nitrated OVA proteins inhibited sensitization probably due to accelerated gastric degradation of nitrated OVA. However, nitrated OVA proteins significantly increased the mediator release of rat basophil leukemia (RBL) cells, passively sensitized with serum of allergic mice. These data indicated that nitrated food proteins influenced the effector phase of allergy, in which the so-called effector cells, such as mast cells, basophils and eosinophils can be activated by the cross link of allergen specific IgE on their surface and can lead to a mediator release, such as histamine, which then mediates the allergic response. If the effector phase was affected by nitration of food allergens it might have a consequences on the severity of food adverse responses [6].

In the current study, the influence of nitration of the milk allergen BLG, a major cow’s milk allergen, and OVM, a major hen’s egg allergen, on the anaphylactic response was evaluated *in vivo* in an experimental mouse model. Additionally, we aimed to analyze the effect of nitration of these food allergens on their structural properties.

## Materials and Methods

### Reagents and animals

Beta-Lactoglobulin (BLG) was purchased from Sigma (Vienna, Austria; purity ≥90%). Ovomucoid (OVM) was purchased from Worthington Biochemical Corporation (Lakewood NJ, USA). Enzyme-linked immunosorbent assay (ELISA) for mMCP-1, IL-4, IFN-γ and IL-10 were purchased from eBioscience (Vienna, Austria).

Female BALB/cAnNCrl mice (n = 48, aged 6–8 weeks, 15–20 g, provided with a health report certificate) were purchased from the Core Unit for Biomedical Research, Division for Laboratory Animal Science and Genetics (Himberg, Austria) and housed under conventional conditions (12 h light/dark cycle at 22°C) in groups of 5 in polycarbonate Makrolon type II cages (Ehret GmbH, Emmendingen, Germany) with filter tops and espen wood bedding (Ehret GmbH, Emmendingen, Germany) enriched with nesting material of cellulose and red transparent plastic nest boxes. Mice were kept on an egg and cow’s milk free diet (Ssniff, Soest, Germany) with *ad libitum* access to food and water. After an acclimation period of 2 weeks all experimental procedures were carried out per group in the morning in a separate animal experimentation room in random order regarding animals within each group. Sample size calculation was based on previous own data [20] with a power calculation based on two-sided two sample T-test. The concept of 3Rs (replacement, refinement and reduction) had a fundamental impact on study design of the approved ethical protocol. Primary outcome of the animal study was to investigate the influence of nitrated BLG and OVM on anaphylactic responses indicated by drop of core body temperature and elevated mMCP1 levels. Secondary experimental endpoints were defined as influences on antibody titers and T-cell response.

### Ethics Statement

Animals were treated according to European Union guidelines of animal care. The protocol was approved by the ethics committee of the Medical University of Vienna and the Austrian Federal Ministry of Science and Research (permission number GZ BMWF-66.009/0270-II/3b/2013).

### Nitration of BLG and OVM

Beta-lactoglobulin and OVM were nitrated or sham-nitrated as previously described [6,7]. Briefly, allergens were nitrated using tetranitromethane (TNM) (Sigma, Vienna, Austria) diluted in methanol (Merck, Darmstadt, Germany) for 60 min. Sham-nitration of allergens was done accordingly without adding the nitrating reagent TNM. After sham-treatment or nitration, reaction was stopped by buffer exchange. Samples were centrifuged through a 10 kDa ultrafiltration tube (Merck Millipore, Cork, Ireland) to remove the reagents (residual MeOH and TNM), at 4000 rpm for 10 min. This step was repeated twice with fresh phosphate buffer (10 mM, pH 7.45). Protein concentrations were determined by amino acid analysis and Bradford assays. The degree of nitration was assessed according to a 3-NT standard curve ranging from 5–200 μmol/l by measuring absorbance at 428–650 nm.

### Immunization regimen

According to internationally established protocols [21,22], mice were immunized i.p. with untreated BLG (n = 15) or OVM (n = 15) (2 μg protein adsorbed to 2 mL aluminium hydroxide) (Alu-Gel-S, Serva Electrophoresis, Heidelberg, Germany) for 3 times in 14 day intervals. Mice were twice challenged orally with untreated BLG or OVM (50 mg per mouse in PBS) and core body temperature was measured before, 15 and 30 min after oral challenge (OC).

**Fig 1:**
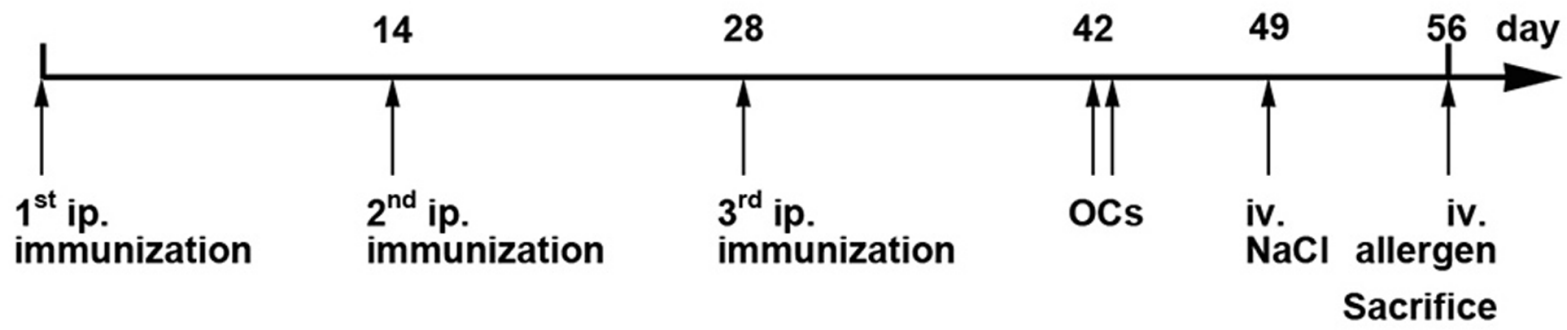
Immunization protocol. Mice were immunized i.p. with untreated BLG (n = 15) or OVM (n = 15) three times, followed by two oral challenges (OCs) on days 42 and 43. Subsequently, mice were divided in three groups per allergen (n = 5) and challenged i.v. with vehicle control on day 49 followed by i.v allergen challenges on day of sacrifice. Blood samples were collected on days 42, 49 and 56. BLG, beta-lactoglobulin; i.v., intravenous; OC, oral challenge; OVM, ovomucoid

One week before sacrifice, mice were injected 0.9% sodium chloride and rectal temperature was measured to rule out an unspecific drop of temperature after i.v. injection. On day of sacrifice, BLG and OVM allergic animals, respectively, were randomly allocated to 3 groups of allergic mice (n = 5, except for allergic OVMs: n = 4) and challenged i.v. either with untreated (BLG; OVM), sham-nitrated (BLGs; OVMs) or nitrated (BLGn; OVMn) BLG or OVM (50 μg per mouse solved in 0.9% NaCl solution). Temperature was measured before and 15 and 30 min after i.v. challenge using a Thermalert TH-5 thermometer (Physitemp Instruments Inc., Clifton, NJ, USA) to monitor anaphylactic responses. One hour after i.v. challenge, mice were sacrificed and blood was taken by cardiac puncture for mMCP-1, IgE, IgA, IgG1 and IgG2a detection and RBL assays. For control purpose, naïve animals (n = 18) were left untreated during the sensitization period. On the day of sacrifice, naïve animals were randomly divided into 6 groups (n = 3) and were i.v. challenged either with untreated (BLG; OVM), sham-nitrated (BLGs; OVMs) or nitrated (BLGn; OVMn) BLG or OVM, respectively (Fig 1). Blood sampling was done before the first immunization, on days 43 and 49 from the tail vein and at sacrifice by cardiac puncture. Allergic and naïve groups were named according to the i.v. allergen challenge (BLG/OVM, BLGs/OVMs and BLGn/OVMn). Samples collected prior to the final random allocation of mice were afterwards designated to the respective group and named likewise.

### Detection of allergen specific antibodies

Mouse serum samples were screened for BLG and OVM specific IgG1, IgG2a, IgA and IgE as described recently [20]. Briefly, serum samples were diluted 1:20 for IgE and 1:100 for IgG1, IgG2a and IgA in blocking buffer (TBS containing 0.05% Tween (TBST) and 1% BSA). Detection was performed with rat anti-mouse IgG1, IgG2a, IgA and IgE (all from BD Biosciences, Heidelberg, Germany) were diluted 1:500 in TBS containing 0.05% Tween and 0.1% BSA being followed by peroxidase labeled goat anti-rat IgG (Amersham, Buckinghamshire, UK; 1:1000 in TBST/0.1%BSA). Tetramethylbenzidine (TMB; BD Bioscience) was used as substrate and reaction was stopped with 0.8mol/L H_2_SO_4_. Antibody titers were calculated according to a standard dilution series ranging from 100 to 0.78 ng/mL for IgE and ranging from 400 to 3.13 ng/mL for all other isotypes.

### RBL-Assay

Rat basophil leukemia cells (RBL-2H3 cells), which bind murine IgE via the high affinity IgE receptor FcεRI, were used to determine the binding and crosslinking ability of the different allergen formulations [23,24]. Cells were cultivated in DMEM (Gibco Life Technologies, Vienna, Austria) supplemented with 10% fetal bovine serum (FBS; v/v) (Gibco Invitrogen, Vienna, Austria), 1% Pen/Strep (Gibco Life Technologies, Vienna, Austria), 1% Glutamine (200 mM, Gibco Life Technologies, Vienna, Austria), 1% non-essential amino acids (Gibco Life Technologies, Vienna, Austria) and 1% HEPES (Gibco Life Technologies, Vienna, Austria). 4x10^4^ cells/well were plated in round bottom cell culture plates and incubated overnight at 37°C. Cells were passively sensitized with serum samples of allergic mice (final dilution 1:20) for 2 h at 37°C to enable binding of BLG or OVM specific IgE to the receptor. Untreated, sham-nitrated and nitrated BLG and OVM (10 μg/ml) were incubated to sensitized RBL-cells in triplicates, respectively, for 30 min. For 100% release, Triton X-100 (Bio-Rad, Vienna, Austria) (10%; v/v) was added as positive control for 5 min. Mediator release was evaluated indirectly in supernatants by measuring the activity of β-hexosaminidase after addition of 4-Methylumbelliferyl N-acetyl-β-D-galactosaminide (4-MUG; Sigma, Vienna, Austria) as substrate. After 1 h incubation time, the reaction was stopped using glycine buffer (pH 10.7) and fluorescence of the formed products was measured at 360nm- 465nm. Results were presented relative to the positive control.

### Detection of mMCP-1

Mouse MCP-1 was measured using the ELISA kit (eBioscience) according to manufacturer’s instructions and as described recently [25]. Briefly, microtiter plates were coated with capture antibody overnight. After blocking, standards and serum samples (dilution 1:100) were added for overnight incubation at 4°C. After incubation with a detection antibody and avidin-horseradish peroxidase (HRP), TMB was added and the color reaction was measured at 450–570 nm. Mouse MCP-1 levels were calculated according to a standard curve ranging from 15 to 0.23 ng/mL.

### Cytokine determination

Spleens were removed under sterile conditions, minced and filtered using sterile nylon cell strainers (40 μm). Erythrocytes were lysed by addition of 5 ml ACK lysing buffer (Lonza, Basel, Switzerland) for 5 min. Cells were washed two times in RPMI containing 10% FCS (v/v) and counted using an automated Coulter Counter (TC10; Bio-Rad). 4 x 10^5^ cells per well were incubated in triplicate with 2.5 μg/ml of untreated, sham-nitrated or nitrated BLG and OVM, respectively, for 72 h at 37°C. Supernatants were then screened for IL-4, IFN-γ and IL-10 levels. ELISAs were performed following the manufacturer’s instructions. Microtiter plates were coated overnight at 4°C with capture antibody, blocked and then incubated with samples (dilution 1:2 in blocking buffer) and standards overnight. After addition of detection antibody and avidin HRP, plates were incubated with TMB and the color reaction was measured at 450–570 nm. Concentrations were determined according to a standard curve ranging from 500 to 3.9 pg/mL for IL-4, from 4000 to 62.5 pg/mL for IL-10 and from 2000 to 15.63 pg/mL for IFN-γ.

### Circular dichroism analysis

Analysis of secondary structure of BLG and OVM (both untreated, sham-nitrated and nitrated) was performed by Far UV Circular Dichroism (CD) spectroscopy as previously described [26].

In short, samples were analyzed using a J-715 spectropolarimeter (Jasco, Gross-Umstadt, Germany) at 0.1 mg/ml diluted in ultrapure distilled H_2_O (BLGs (1/21.55), BLGn (1/14.98), OVMs (1/16.20), OVMn (1/17.84)) in a 1 mm path length quartz cuvette (Hellma, Müllheim, Germany) equilibrated at 20°C. Spectra were recorded at a scan speed of 50 nm/min and a resolution of 0.2 nm. The average of three independent scans was used and after subtraction of the baseline spectrum, results were calculated as mean residue ellipticity (θ) at the respective wavelengths and analyzed using dichroweb program [27,28]. Analysis of CD data in terms of secondary structure was performed by the listed algorithms provided by CDPro (http://lamar.colostate.edu/~sreeram/CDPro/main.html) using the model protein set 7 (SDP48).

### Statistical analysis

Data were statistically analyzed with GraphPad Prism version 5.00 for Windows (GraphPad Software, San Diego California USA, www.graphpad.com) comparing groups as they were defined on day of sacrifice. All available samples were included in the analysis. A single set of experiments was performed as recommended by the current effective animal ethic’s regulations. Samples were checked for Gaussian distribution by applying Kolmogorov-Smirnov normality test. Groups were compared with one-way ANOVA followed by Tukey multiple comparison test or two-way ANOVA combined with Bonferroni post test. A P value < 0.05 was considered statistically significant.

## Results

### Immunization with OVM and BLG induced anaphylactic antibodies

To evaluate the impact of protein nitration on the anaphylactic response of food allergic mice, animals were sensitized by i.p. injections of untreated BLG and OVM adsorbed to aluminium hydroxide followed by two oral challenges with untreated BLG and OVM to induce a strong systemic and local immune response. On day of sacrifice mice were challenged i.v. with untreated (BLG; OVM), sham-nitrated (BLGs; OVMs) or nitrated allergens (BLGn; OVMn) (Fig 1).

**Fig 2:**
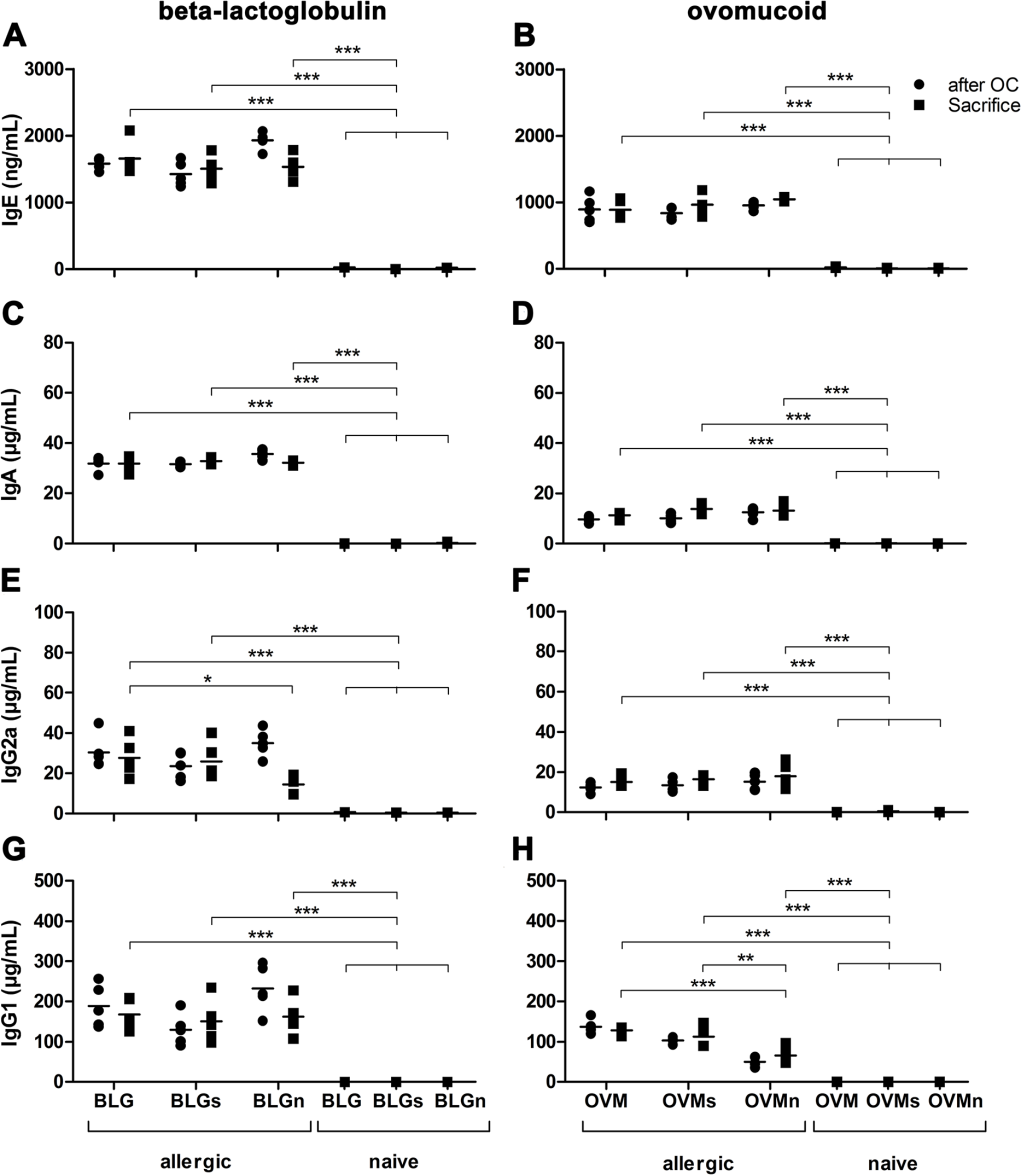
Induction of allergen specific IgE, IgA, IgG1 and IgG2a in immunized mice. Immunization elicited equally elevated specific IgE titers in mice being injected with the same allergen (A, BLG; B, OVM) whereas naïve control groups did not show any allergen specific IgE antibody production. BLG (C, E, G) and OVM (D, F, H) allergic mice showed significantly higher levels of allergen specific IgG1, IgG2a and IgA compared with naïve mice. Significantly lower levels of specific IgG2a were observed in the group i.v. challenged with nitrated BLG (BLGn). Significantly lower levels of specific IgG1 were found in the group i.v. challenged with nitrated OVM (OVMn). Independent of the isotype, higher antibody levels were found in mice sensitized with BLG compared to OVM sensitized animals. Data obtained for IgG2a of OVM allergic mice were transformed by applying the logarithm to base 10. Antibody levels of groups were compared at the time-point of sacrifice with One-way ANOVA followed by Tukey post test. (**P<0.01, ***P<0.001) BLG, beta-lactoglobulin; BLGn, nitrated BLG; i.v., intravenous; OVM, ovomucoid; OVMn, nitrated OVM

The intensity of the immune response after sensitization was evaluated by measuring BLG and OVM specific IgE, IgG1, IgG2a and IgA antibody levels. Beta-lactoglobulin and OVM sensitized mice had significantly elevated allergen specific IgE levels compared to naïve animals (Fig 2A and 2B). No statistical differences were observed between groups immunized with the same allergen. Biological functionality of these IgE antibodies was assessed by RBL-assay using serum samples of BLG and OVM allergic groups collected after oral challenge, after i.v. control challenge with sodium chloride and after i.v. allergen challenge (S1 Fig). RBL-cells were stimulated with untreated, sham-nitrated and nitrated BLG or OVM. Despite the fact that no change of IgE levels was detected over time, statistical comparison revealed the time-point of serum sample collection to significantly influence the levels of mediator release in the groups systemically challenged with untreated, sham-nitrated or nitrated BLG (two-way ANOVA; p<0.0001 BLG, BLGn; p<0.05 BLGs). Stimulation with different formulations of the allergens (untreated, sham-nitrated, or nitrated) did not induce differences in β-hexosaminidase release. Interestingly, a higher β-hexosaminidase release was observed in OVM treated groups compared to BLG sensitized groups, although IgE serum levels were lower.

After the sensitization regimen serum samples were screened for BLG and OVM specific IgA, IgG2a and IgG1 (Fig 2C–2H). Independent of the isotype, higher antibody levels were found in mice sensitized with BLG compared to OVM sensitized animals. All immunized animals showed significantly elevated titers of allergen specific IgA, IgG2a and IgG1 compared to naïve mice. BLG immunization led to significantly lower IgG2a levels in BLGn mice compared to BLGs and BLG mice at the time of sacrifice. In the same group (BLGn) we additionally observed significantly increased levels of IgG2a after oral challenges compared to time-point of sacrifice (p = 0.0004). OVM sensitization led to significantly lower levels of specific IgG1 in OVMn mice compared to OVM and OVMs mice.

### No differences in cytokine response were detectable between the different groups

Supernatants of stimulated splenocytes isolated from allergic mice at the time-point of sacrifice were screened for IL-4, IFN-γ and IL-10 to investigate T-cell responses provoked by BLG (S1 Table) and OVM (S2 Table). Stimulation with BLG did not induce significant differences in cytokine production comparing the different groups. IL-4 levels were mostly below detection limit. Supernatants of OVM treated cells revealed similar mean levels of IL-4, IFN-γ and IL-10 in all OVM immunized groups. For naïve groups, almost no cytokine production could be detected after OVM stimulation, which is in contrast to our findings for BLG stimulation. Conversely, OVM induced detectable levels of IL-4 in sensitized groups, which was not seen for BLG. However, comparison of all immunized groups did not reveal any significant differences between the different groups nor comparing the stimulatory effect of untreated, sham-nitrated or nitrated allergens.

### Nitrated food proteins enhance the anaphylactic response of allergic mice in an allergen dependent manner.

**Fig 3:**
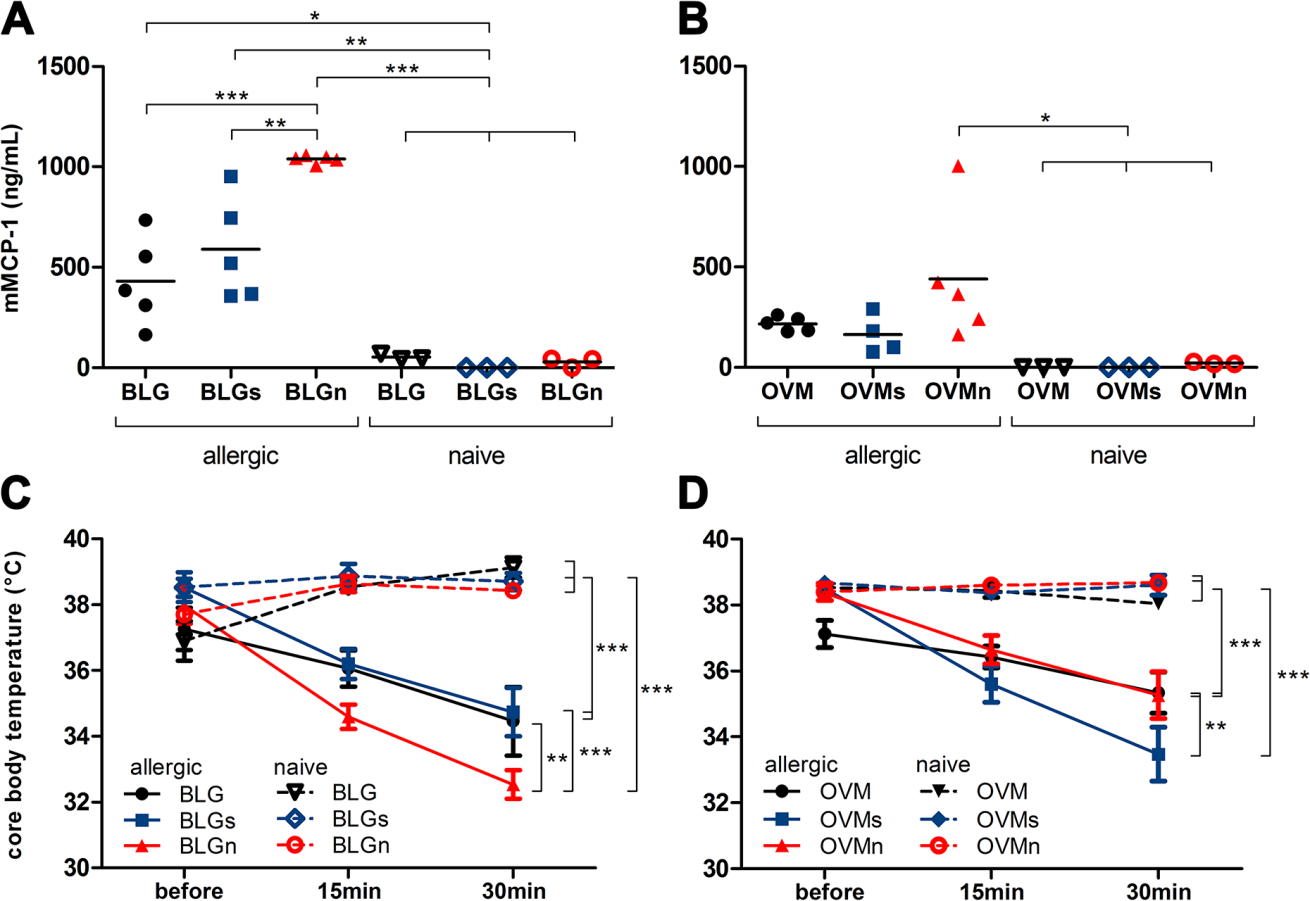
Nitrated BLG enhances the anaphylactic response in BLG immunized animals. After i.v. allergen challenge all BLG immunized groups showed significantly elevated levels of the anaphylaxis marker mMCP-1 compared to naïve control groups (A). Allergic mice injected with nitrated BLG (BLGn) revealed a significantly higher mMCP-1 release compared to mice only receiving untreated (BLG) or sham-nitrated (BLGs) BLG. Systemic challenge of allergic mice with different preparations of OVM did not result in differences of mMCP-1 between OVM, OVMs and OVMn (B). Mouse MCP-1 concentrations were compared with One-way ANOVA. Body temperature of all groups was assessed before and 15 and 30 min after i.v. challenge (C, D). A significant drop of body temperature was observed in all allergic mice. Animals challenged with BLGn showed a significant decline of body temperature compared to BLG and BLGs, while in OVM allergic mice decrease was most prominent in OVMs. Mean temperatures of all groups at 30 min were compared using One-way ANOVA prior to Tukey multiple comparison test (*P>0.05, **P<0.01, ***P<0.001). BLG, beta-lactoglobulin; BLGn, nitrated BLG; BLGs, sham-nitrated BLG; i.v., intravenous; mMCP-1, mouse mast cell protease-1; OVM, ovomucoid; OVMn, nitrated OVM; OVMs, sham-nitrated OVM

To assess the effect of nitration on anaphylactic responses in sensitized mice, animals were challenged i.v. with untreated, sham-nitrated or nitrated BLG or OVM. The systemic response was evaluated in allergic animals by measuring mMCP-1 levels and core body temperature (Fig 3) and compared to naïve animals.

Significantly elevated levels of mMCP-1 were observed in all BLG sensitized animals compared to naïve mice after i.v. challenge with the respective allergen formulation (Fig 3A). Only in BLG mice challenged with nitrated BLG, a significantly higher release of mMCP-1 was found compared to mice challenged with untreated or sham-nitrated BLG. Notably, provocation of OVM allergic animals with the respective OVM preparations did not result in significant changes of mMCP-1 levels between the different allergic mouse groups (Fig 3B).

Core body temperature before, as well as 15 and 30 minutes after i.v. allergen challenge was assessed to objectify anaphylactic responses. All sensitized animals revealed a significant reduction of body temperature compared to naïve, i.v. challenged animals. However, only BLG sensitized mice, receiving nitrated BLG i.v. had a significant decline of body temperature (P<0.05) compared to allergic animals i.v. challenged with untreated or sham-nitrated BLG (Fig 3C). These patterns were not observed in OVM allergic mice being challenged with untreated, sham-nitrated or nitrated OVM preparations, as here OVMs challenge induced a significant drop of body temperature compared to the other allergen formulations (Fig 3D). Injection of vehicle control one week before sacrifice did not influence core body temperature (S2 Fig).

### Enhanced oligomerization and altered secondary protein structure of nitrated BLG but not of OVM

**Fig 4:**
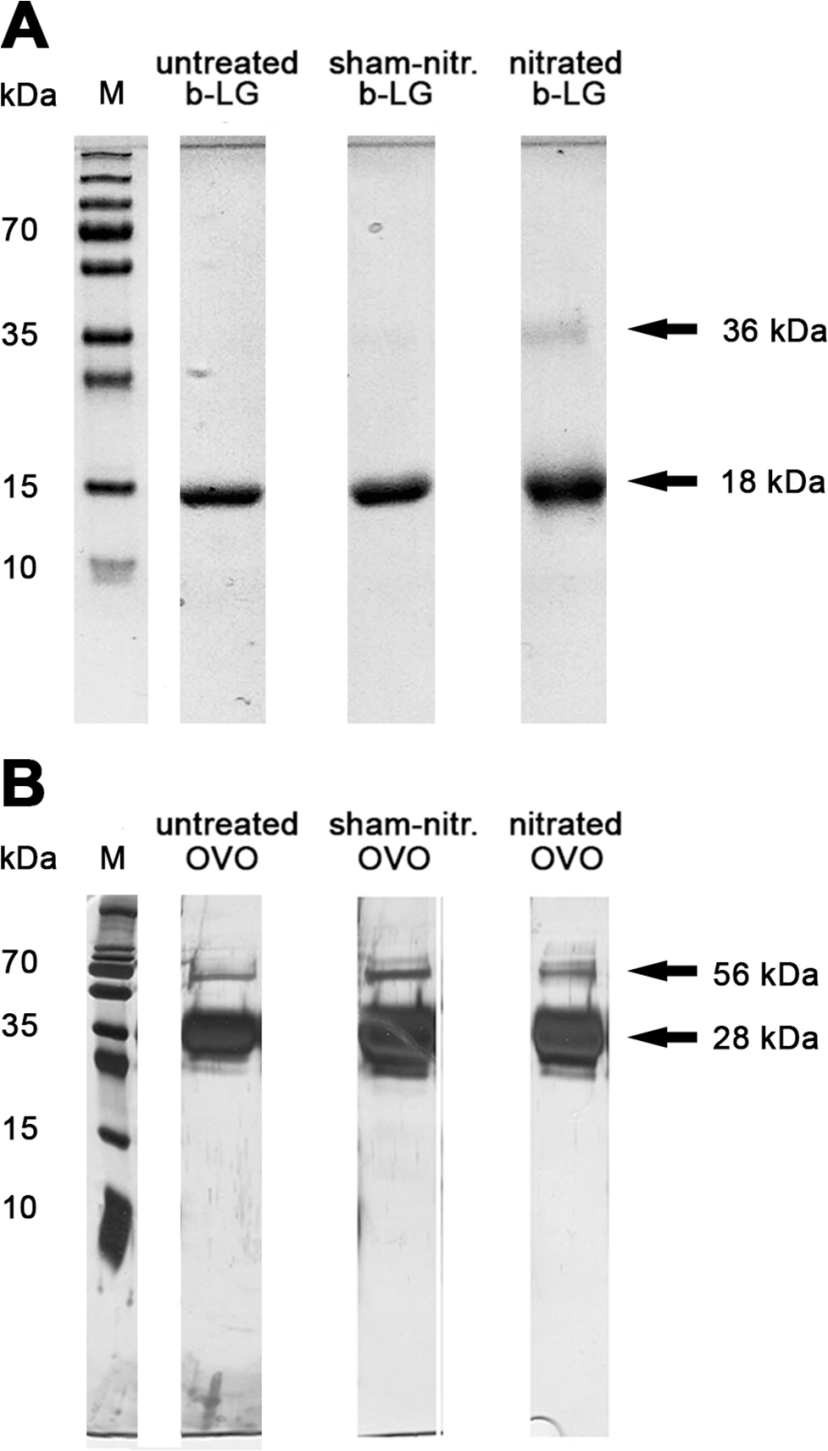
Increased dimerization of nitrated BLG. (A) SDS-PAGE analysis revealed an enhanced dimerization of nitrated BLG compared to untreated and sham-nitrated proteins. (B) OVM formed dimers independent of the nitration status. BLG, beta-lactoglobulin; M, marker lane; OVM, ovomucoid

The effect of protein nitration on oligomerization was evaluated by SDS-PAGE analysis (Fig 4). Interestingly, nitration of BLG, which naturally forms dimers at pH above 3 [29] was associated with an enhanced oligomer formation compared to untreated and sham-nitrated BLG (Fig 4A). OVM exhibited formation of dimers independent of the nitration status, as untreated, sham-nitrated and nitrated OVM showed comparable degrees of protein dimerization in SDS-PAGE (Fig 4B).

**Fig 5:**
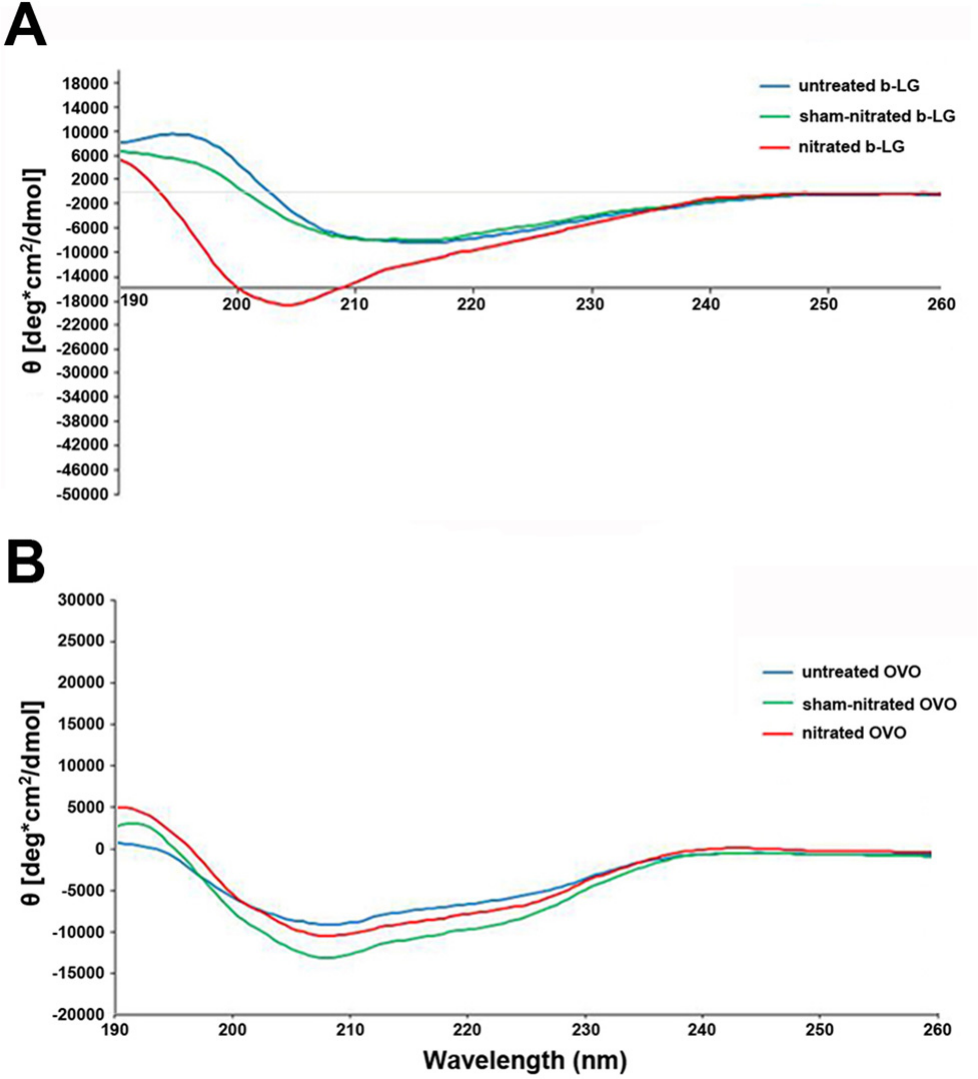
Nitrated BLG reveals an altered secondary structure. Samples originally dissolved in 10 mM Na_2_HPO_4_ buffer were diluted to 0.1 mg/ml in ultrapure distilled water according to the following ratios for: BLGs (1/21.55), BLGn (1/14.98), OVMs (1/16.20), OVMn (1/17.84). For nitrated BLG (A) an alteration of the secondary structure was observed as indicated by changed spectra, which was not observed for OVM (B). Results are expressed as the mean residue ellipticity Θ (y-axis) and plotted against the respective wavelength (x-axis). BLG, beta-lactoglobulin; OVM, ovomucoid

Circular dichroism analysis (Fig 5) of untreated, sham-nitrated and nitrated OVM revealed only limited influence of nitration on the secondary protein structure. In contrast, CD spectra indicated substantial changes of the secondary protein structure of nitrated BLG causing a shift in the minima near 200 nm compared to untreated and sham-nitrated proteins (Fig 5A). Nitration of BLG resulted in higher abundance of α-helical structures and unordered domains accompanied by a decline of β-sheets compared to untreated or sham-nitrated BLG (Table 1).

**Table 1:** Circular dichroism analysis.

	β-lactoglobulin			ovomucoid		
Secondary structure	untreated	sham-nitr.	nitrated	untreated	sham-nitr.	nitrated
α-helix	0.199 ± 0.003	0.171 ± 0.003	0.233 ± 0.017	0.162 ± 0.002	0.234 ± 0.008	0.194 ± 0.006
β-sheet	0.304 ± 0.021	0.292 ± 0.015	0.181 ± 0.056	0.206 ± 0.011	0.165 ± 0.041	0.223 ± 0.003
turn	0.216 ± 0.006	0.207 ± 0.005	0.179 ± 0.019	0.174 ± 0.002	0.196 ± 0.025	0.198 ± 0.008
unordered	0.280 ± 0.019	0.330 ± 0.022	0.418 ± 0.030	0.463 ± 0.004	0.411 ± 0.045	0.390 ± 0.013

Protein secondary structure was estimated by the programs CONTIN, SELCON3 and CDSSTR using the model protein set 7 (SDP48). Values calculated for secondary structures by either program were averaged and presented as mean values ± standard deviation, respectively.

## Discussion

The fact that nitration can alter allergenicity of food proteins was described recently [6]. However, as indicated by the data presented here, the increase of allergenicity after nitration is not a common phenomenon of all food allergens but rather is allergen specific. In our study, we investigated the allergenic potential of nitration for two common food proteins: the major cow’s milk allergen BLG and the major hen’s egg white allergen OVM.

BLG is a member of the lipocalin family [30,31] consisting of 162 amino acids (AA) [32] after cleavage of the signal sequence of 16 AA. Each monomer of BLG contains only 4 tyrosine residues and 2 disulfide bonds together with one free cysteine [33] (source: http://www.uniprot.org/uniprot/P02754). OVM consists of 210 AA, whereby 24 AA are from the signal peptide, resulting in a length of 186 AA of the final protein. OVM comprises six tyrosine residues, nine disulfide bonds and five glycosylation sites (source: http://www.uniprot.org/uniprot/P01005). Despite the comparable number of tyrosine residues, our data clearly indicate that only nitrated BLG triggers an enhanced anaphylactic response. We revealed significant reduction of body temperature in all allergic animals after systemic provocation, which was especially pronounced after challenge with nitrated BLG. This effect was not observed with nitrated OVM. These findings are supported by significantly increased levels of the mucosal mast cell marker mMCP1 after i.v. challenge with nitrated BLG. Of interest, we measured significant reduced levels of mediator release in *in vitro* RBL assays using sera collected at time-point of challenge, which was most likely due to vascular leakage during anaphylaxis. The absent decline of clinical reactivity after injection of nitrated OVM to allergic animals might be explained by the structural characteristics of the protein. Sequence analysis (S3 Fig) revealed that OVM has four IgE binding epitopes [34] and two T-cell epitopes [35] the latter comprising a tyrosine residue each. However, according to the model by Cooke and Sampson 1997 [36], these tyrosine residues might not be fully accessible for nitration due to their protected position within the protein’s secondary structure being associated with different degrees of tyrosine residue nitration as previously demonstrated for other molecules [6,12]. This is supported by routine analysis of the nitration degree in our laboratory indicating a 34 fold higher nitration degree for BLG compared to OVM. Additionally, nitration of OVM did not change its tendency for protein dimerization. Observed dimerization of nitrated BLG may be a result of 3,3’-dityrosine formation during the process of nitration when a biphenyl bond is established between two tyrosyl radicals [37]. Aggregation of food allergens would increase stability and resistance to digestion, would favor uptake via Payer’s patches and the underlying immune system [38] and enhance cross-linking of IgE antibodies bound to effector cells of allergy [39] as it has been shown for respiratory allergens [40].

The protein structure of OVM is stabilized by nine disulfide bonds and contributing cysteines are located within a maximal distance of 4 AA to tyrosine residues limiting potential interactions between tyrosines of two chemically altered molecules. Reducing disulfide bonds might be associated with exposure of these residues [41] possibly changing the impact of protein nitration. In comparison, the 4 tyrosine residues of BLG have a different solvent accessibility. While one could speculate that Y42 and Y102 might not be fully accessible for the nitrating agent thus being less extensively nitrated, Y20 is accessible to most solvents and close to the base of the putative binding pocket. Additionally, Y20 is part of an IgG binding epitope spanning AA 22 to 36 [42]. Y99 is exposed on the external loop of the protein and is additionally part of the γ turn, which contains the most highly conserved triplet of residues within the lipocalin family (T97, D98, Y99) [33]. The latter two tyrosine residues are part of T-cell epitopes [43] of which only the Y99 is exposed. Based on this evidence and previous data [6], nitration of food allergens differently affects sensitization and effector phase of food allergy. Probably depending on the solvent accessibility or location of tyrosine residues within the amino acid sequence (i.e. hydrophobic and acidic amino acids in the vicinity of tyrosine), which influences the propensity for nitration, the tested allergen BLG shows specifically altered allergenicity after nitration. In case nitrated tyrosine residues are located within epitopes or when aggregation is favored, nitrated food allergens might enhance the allergic response by increasing the release of mast cell mediators leading to anaphylaxis.

Even though our *in vivo* results are based on mouse experiments with limitations due to known differences compared to human immune response [44], we propose that nitration of specific food allergens might have major impact on the clinical response in food allergic patients. While some allergic patients tolerate small amounts of allergen without an anaphylactic reaction upon ingestion under regular conditions as indicated during double-blinded placebo-controlled food challenges (DBPCFCs) [45], these patients might reveal changed clinical reactivity upon ingestion of nitrated food proteins. It has to be taken into consideration that patients might encounter nitrated allergens not only by ingestion but also during endogenous inflammatory processes associated with food protein nitration [13] or due to production of nitrating species in the stomach [18]. If allergic patients encounter nitrated food allergens it may provoke anaphylactic responses at even lower doses than observed in DBPCFC with untreated allergens.

In this study we report an enhanced anaphylactic capacity of the nitrated food allergen BLG. Therefore, we anticipate that nitrated food allergens may facilitate anaphylactic responses at lower doses than observed during diagnosis of food allergy in DBPCFC with untreated allergens. Therefore, for practical patients´ recommendations it may be highly important to specifically identify those allergens where nitration leads to substantial increase in allergenicity, such as BLG.

## Supporting Information

S1 Fig Immunization induced biologically functional, allergen specific IgE antibodies. Biological functionality of IgE antibodies was assessed by RBL assays. RBL-cells were passively sensitized with serum samples from all allergic groups, which were named according to the following i.v. challenge: untreated BLG (A) or OVM (B), sham-nitrated BLG (BLGs; C) or OVM (OVMs; D) and nitrated BLG (BLGn; E) or OVM (OVMn; F). Samples were collected after oral challenge with untreated allergen (OC), after vehicle injection (i.v. NaCl) or after systemic allergen administration (i.v. allergen). Cells were stimulated with untreated (small squares filled columns), sham-nitrated (bigger squares filled columns) and nitrated (lined columns) BLG (left panels) or OVM (right panels). Data were analyzed with Two-way ANOVA followed by Bonferroni post test. Results are presented as mean values and standard error of the mean (SEM). (*P>0.05, **P>0.01) BLG, beta-lactoglobulin; BLGn, nitrated BLG; BLGs, sham-nitrated BLG; i.v., intravenous; OC, oral challenge; OVM, ovomucoid; OVMn, nitrated OVM; OVMs, sham-nitrated OVM
(TIF)

S2 Fig Intravenous injection of vehicle control does not induce a temperature decline. One week before sacrifice, naïve and allergic mice were injected with 0.9% sodium chloride and rectal temperature was measured to rule out an unspecific drop of temperature after i.v. injection. Neither BLG/OVM allergic nor naïve mice showed a drop of core body temperature within 30 min after i.v. administration of vehicle.
(TIF)

S3 Fig Sequence information on BLG and OVM. BLG (A) consists of 178 AA with 16 AA from the sinal peptide and 162 AA of the final protein including 4 tyrosine residues (yellow) at the positions 20, 42, 99 and 102. OVM (B) comprises 210 amino acids: 24 AA are from the signal peptide and 186 AA form the final protein. Tyrosine residues (labelled yellow) are located at positions 37, 46, 73, 102, 141 and 161. Positions are indicated referring to the final proteins without the signal peptide. T cell, IgE- and IgG- binding epitopes, two disulfide bonds (C marked in cyan) and glycosylation sites (N marked in green) are indicated. AA, amino acid; BLG, beta-lactoglobulin; OVM, ovomucoid
(TIF)

S1 Table Cytokine levels of splenocytes stimulated with untreated BLG. Results are presented as mean values ± SEM. BLG, beta-lactoglobulin
(PDF)

S2 Table Cytokine levels of splenocytes stimulated with untreated OVM. Results are presented as mean values ± SEM. OVM, ovomucoid
(PDF)
