# Supplementary figures and images for: Nitration of β-Lactoglobulin but Not of Ovomucoid Enhances Anaphylactic Responses in Food Allergic Mice
Source: PLoS One. 2015 May 8;10(5):e0126279. doi: 10.1371/journal.pone.0126279 (PMC4425501; doi:10.1371/journal.pone.0126279)

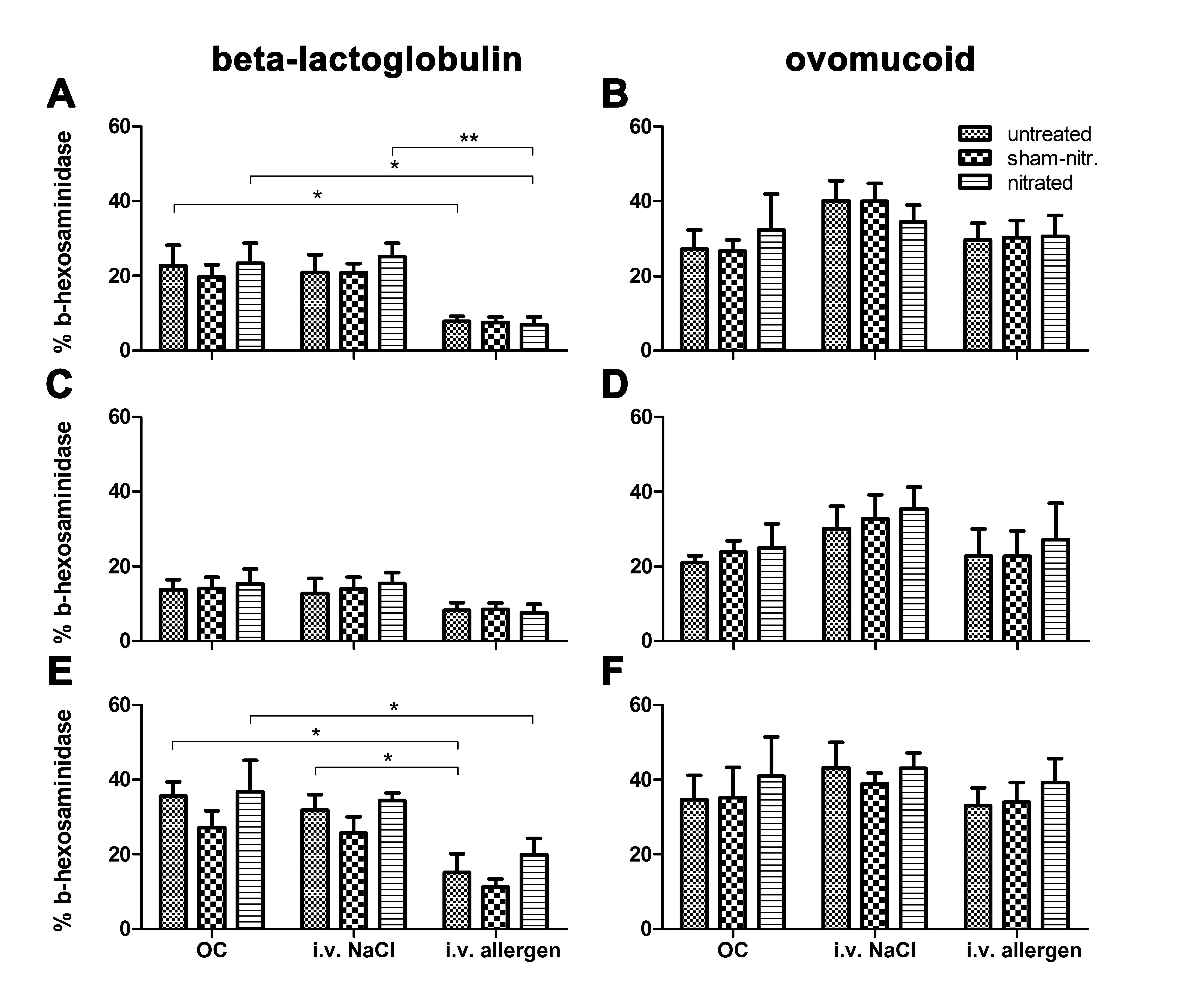

Supplement: S1 Fig — Biological functionality of IgE antibodies was assessed by RBL assays. RBL-cells were passively sensitized with serum samples from all allergic groups, which were named according to the following i.v. challenge: untreated BLG (A) or OVM (B), sham-nitrated BLG (BLGs; C) or OVM (OVMs; D) and nitrated BLG (BLGn; E) or OVM (OVMn; F). Samples were collected after oral challenge with untreated allergen (OC), after vehicle injection (i.v. NaCl) or after systemic allergen administration (i.v. allergen). Cells were stimulated with untreated (small squares filled columns), sham-nitrated (bigger squares filled columns) and nitrated (lined columns) BLG (left panels) or OVM (right panels). Data were analyzed with Two-way ANOVA followed by Bonferroni post test. Results are presented as mean values and standard error of the mean (SEM). (*P>0.05, **P>0.01) BLG, beta-lactoglobulin; BLGn, nitrated BLG; BLGs, sham-nitrated BLG; i.v., intravenous; OC, oral challenge; OVM, ovomucoid; OVMn, nitrated OVM; OVMs, sham-nitrated OVM (TIF) [file pone.0126279.s001.tif]

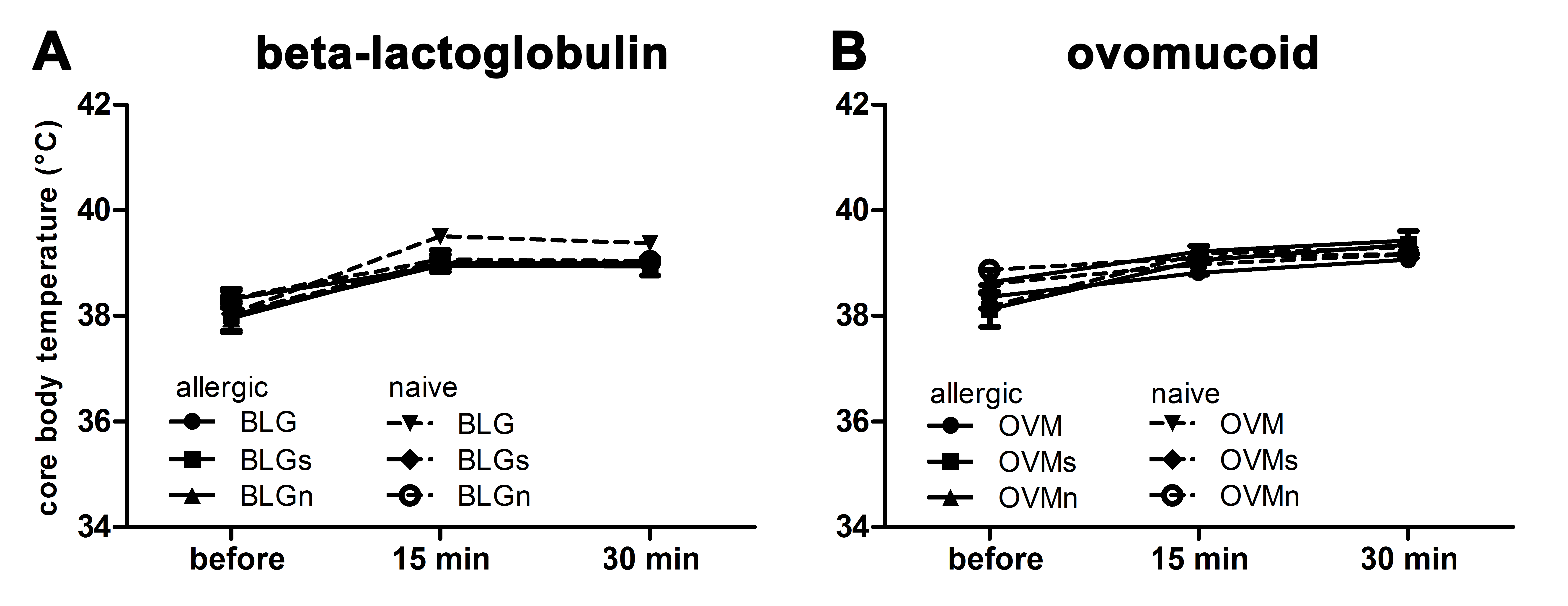

Supplement: S2 Fig — One week before sacrifice, naïve and allergic mice were injected with 0.9% sodium chloride and rectal temperature was measured to rule out an unspecific drop of temperature after i.v. injection. Neither BLG/OVM allergic nor naïve mice showed a drop of core body temperature within 30 min after i.v. administration of vehicle. (TIF) [file pone.0126279.s002.tif]

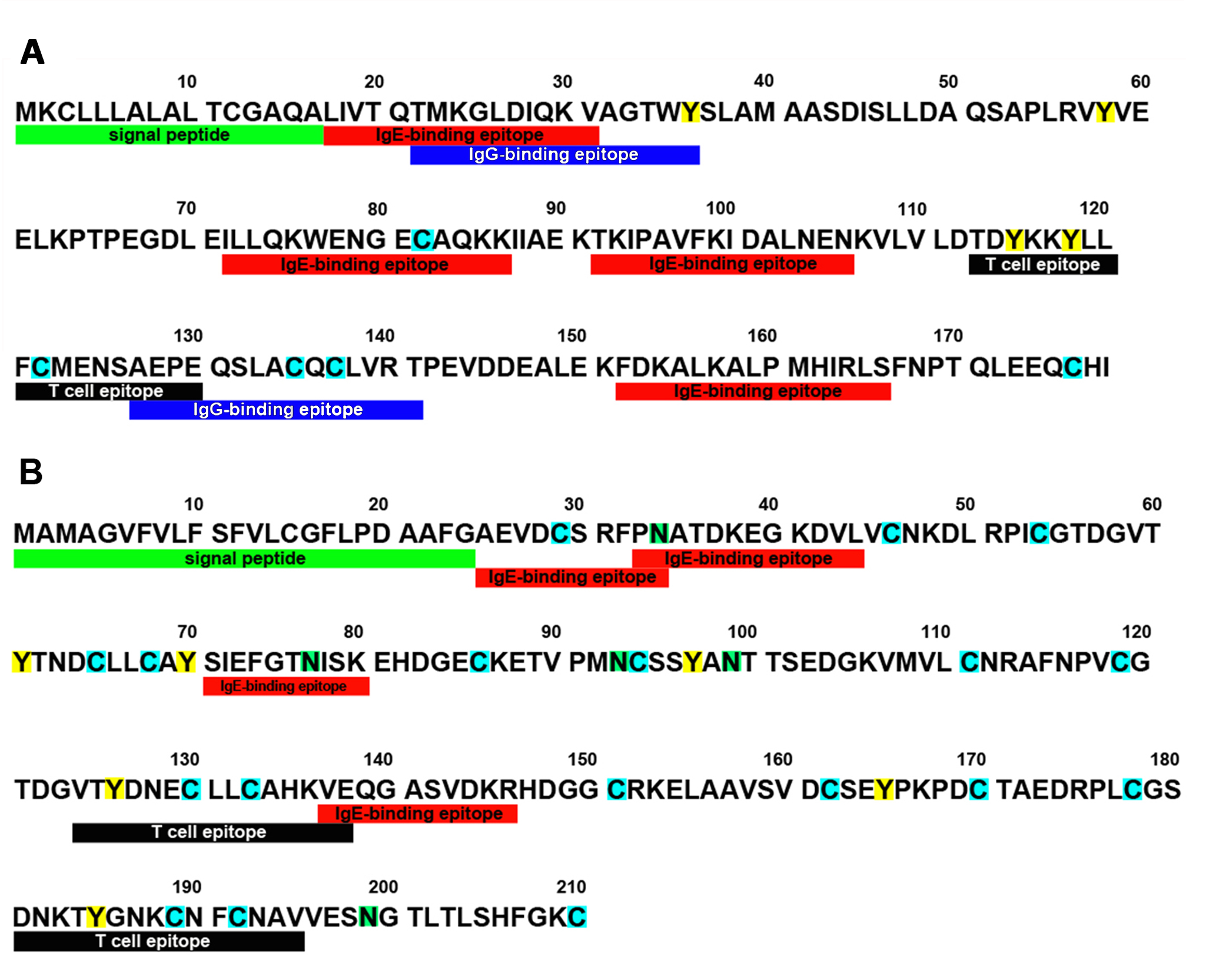

Supplement: S3 Fig — BLG (A) consists of 178 AA with 16 AA from the sinal peptide and 162 AA of the final protein including 4 tyrosine residues (yellow) at the positions 20, 42, 99 and 102. OVM (B) comprises 210 amino acids: 24 AA are from the signal peptide and 186 AA form the final protein. Tyrosine residues (labelled yellow) are located at positions 37, 46, 73, 102, 141 and 161. Positions are indicated referring to the final proteins without the signal peptide. T cell, IgE- and IgG- binding epitopes, two disulfide bonds (C marked in cyan) and glycosylation sites (N marked in green) are indicated. AA, amino acid; BLG, beta-lactoglobulin; OVM, ovomucoid (TIF) [file pone.0126279.s003.tif]
