# Supplementary material for: Nitration of β-Lactoglobulin but Not of Ovomucoid Enhances Anaphylactic Responses in Food Allergic Mice
Source: PLoS One. 2015 May 8;10(5):e0126279. doi: 10.1371/journal.pone.0126279 (PMC4425501; doi:10.1371/journal.pone.0126279)
Supplement: S1 Table — Results are presented as mean values ± SEM. BLG, beta-lactoglobulin (PDF) [file pone.0126279.s004.pdf]

S1 Table. Cytokine levels of splenocytes stimulated with untreated BLG

**Group**	**IL-4**	**IFN-γ**	**IL-10**
1	0	115.650 ± 83.963	224.525 ± 43.274
2	0	434.800 ± 243.019	157.175 ± 61.429
3	0	129.400 ± 86.038	236.500 ± 65.238
4	0	138.958 ± 87.654	142.167 ± 142.167
5	0	165.292 ± 165.292	329.083 ± 135.513
6	4.877 ± 2.814	4.877 ± 2.814	192.429 ± 130.138

Results are presented as mean values ± SEM. BLG, beta-lactoglobulin
