# Supplementary material for: Nitration of β-Lactoglobulin but Not of Ovomucoid Enhances Anaphylactic Responses in Food Allergic Mice
Source: PLoS One. 2015 May 8;10(5):e0126279. doi: 10.1371/journal.pone.0126279 (PMC4425501; doi:10.1371/journal.pone.0126279)
Supplement: S2 Table — Results are presented as mean values ± SEM. OVM, ovomucoid (PDF) [file pone.0126279.s005.pdf]

S2 Table. Cytokine levels of splenocytes stimulated with untreated OVM

**Group**	**IL-4**	**IFN-γ**	**IL-10**
1	38.908 ± 13.568	143.550 ± 78.223	176.920 ± 65.532
2	31.420 ± 18.048	118.389 ± 118.389	63.317 ± 30.811
3	42.297 ± 9.020	110.511 ± 32.973	171.833 ± 45.956
4	4.643 ± 4.643	0	0
5	0	0	0
6	0	0	120.556 ± 120.556

Results are presented as mean values ± SEM. OVM, ovomucoid
